# Effects of SGLT2-Inhibitors on Comprehensive Geriatric Assessment, Biomarkers of Oxidative Stress, and Platelet Activation in Elderly Diabetic Patients with Heart Failure with Preserved Ejection Fraction

**DOI:** 10.3390/ijms25168811

**Published:** 2024-08-13

**Authors:** Marcello Magurno, Velia Cassano, Francesco Maruca, Carlo Alberto Pastura, Marcello Divino, Federica Fazio, Giandomenico Severini, Elvira Clausi, Giuseppe Armentaro, Sofia Miceli, Raffaele Maio, Egidio Imbalzano, Francesco Andreozzi, Marta Letizia Hribal, Angela Sciacqua

**Affiliations:** 1 Department of Medical and Surgical Sciences, University Magna Græcia of Catanzaro, 88100 Catanzaro, Italy; mmagurno14@gmail.com (M.M.); velia.cassano@unicz.it (V.C.); francescomaruca28@gmail.com (F.M.); carloalbertopastura@gmail.com (C.A.P.); marcydivino1987@gmail.com (M.D.); federica.fazio@tiscali.it (F.F.); giandomenicoseverini@gmail.com (G.S.); elvira.clausi@gmail.com (E.C.); andreozzif@unicz.it (F.A.); hribal@unicz.it (M.L.H.); sciacqua@unicz.it (A.S.); 2 Geriatric Division, University Hospital “Renato Dulbecco”, 88100 Catanzaro, Italy; sofy.miceli@libero.it (S.M.); raf_maio@yahoo.it (R.M.); 3 Department of Clinical and Experimental Medicine, Polyclinic University of Messina, 98122 Messina, Italy; egidio.imbalzano@unime.it; 4 Research Center for the Prevention and Treatment of Metabolic Diseases (CRMETDIS), University “Magna Graecia” of Catanzaro, 88100 Catanzaro, Italy

**Keywords:** SGLT2i, T2DM, cognitive impairment, elderly, oxidative stress

## Abstract

Background: Heart failure (HF) with preserved ejection fraction (HFpEF) represents a major comorbidity in the elderly and is associated with cognitive impairment (CoI) and type 2 diabetes mellitus (T2DM). In this context, there is an increase in oxidative stress and platelet activation biomarkers. The aim of this study was to evaluate the effects of 6 months’ treatment with SGLT2i on functional, mood-related, and cognitive aspects, assessed by performing a comprehensive geriatric assessment (CGA), and on oxidative stress and platelet activation biomarkers, in a cohort of HFpEF elderly patients with T2DM. We recruited 150 elderly outpatients (mean age 75.8 ± 7.4 years). Results: At six-month follow-up, there was a significant improvement in MMSE (*p* < 0.0001), MoCA (*p* < 0.0001), GDS score (*p* < 0.0001), and SPPB (*p* < 0.0001). Moreover, we observed a significant reduction in Nox-2 (*p* < 0.0001), 8-Isoprostane (*p* < 0.0001), Sp-Selectin (*p* < 0.0001), and Gp-VI (*p* < 0.0001). Considering ΔMMSE as the dependent variable, ΔE/e’, ΔNox-2, ΔHOMA, Δ8-Isoprostane, and ΔUricemia were associated for 59.6% with ΔMMSE. When ΔMoCA was considered as the dependent variable, ΔHOMA, ΔE/e’, Δ8-Isoprostane, ΔNox-2 and ΔUricemia were associated for 59.2%. Considering ΔGDS as the dependent variable, ΔHOMA, ΔNox-2, Δ8-Isoprostane, and ΔUricemia were associated with 41.6% of ΔGDS variation. Finally, ΔHOMA was the main predictor of ΔSPPB, which was associated with 21.3% with ΔSPPB, Δ8-Isoprostane, ΔNox-2, ΔE/e’, and ΔUricemia added another 24.1%. Conclusion: The use of SGLT2i in elderly patients with T2DM and HFpEF significantly contributes to improving CGA scales and biomarkers of OS and PA.

## 1. Introduction

### 1.1. Epidemiology

Heart failure (HF) with preserved ejection fraction (HFpEF) is the most common type of HF in elderly subjects and is associated with poor quality of life (QoL) and increased mortality. In addition, HFpEF is associated with several comorbidities, such as atrial fibrillation (AF), chronic kidney disease, cognitive impairment (CoI), and type 2 diabetes mellitus (T2DM), complicating their clinical management [1,2]. About 40% of HF patients have cognitive impairment (CoI) and depressive symptoms; this percentage is even higher in individuals with NYHA Class III–IV; in fact, decreased heart function is independently associated with deterioration in many cognitive domains [3].

### 1.2. Interconnection between Heart Failure, Type 2 Diabetes Mellitus, and Cognitive Impairment

Hemodynamic alterations with consequent cerebral hypoperfusion may play a crucial role in the interconnection between HF, CoI, and depressive symptoms [4,5]. CoI is widely recognized as a specific determinant of chronic disability with impairment in domains of both verbal and visual memory, attention, and executive functions [3,6,7,8,9,10,11]. Mild CoI has a negative effect on the therapeutic management of HF as it reduces adherence to drug treatment and worsens QoL and clinical prognosis [12,13]. According to this, HF patients with concomitant CoI are characterized by a 5-fold increased mortality risk [14]. In addition to CoI, T2DM also represents one of the most frequent comorbidities in HFpEF patients and increases the risk of CoI, depression, and reduced QoL [15]. In fact, T2DM and insulin resistance are associated with age-related cognitive decline, increased levels of β-amyloid peptide, phosphorylation of tau protein, and oxidative stress [16,17]. The increase of oxidative stress and platelet activation biomarkers in HF and T2DM, besides being an expression of RAA hyperactivity, may affect clinical outcomes, and at the same time, they may represent possible therapeutic targets [18]. 

### 1.3. Role of Comprehensive Geriatric Assessment

In this contest, the comprehensive geriatric assessment (CGA) is a useful, multidimensional, and interdisciplinary tool to assess cognitive and functional abilities as well as depressive symptoms in different clinical domains of elderly HFpEF patients. Indeed, it is known that CGA is able to predict short- and long-term mortality in patients hospitalized for HF. However, the association between HF and CoI, which recognizes multifactorial mechanisms, is not fully elucidated. Previous evidence showed that hemodynamic alterations with consequent cerebral hypoperfusion and vascular damage may play a crucial role in the interconnection between HF and CoI [19,20]. 

### 1.4. Impact of SGLT2-Inhibitors on Cognitive Impairment

Sodium-glucose cotransporter 2 inhibitors (SGLT2i) lower blood glucose levels by inhibiting SGLT2 in the renal tubules and have been shown to reduce cardiovascular (CV) risk in subjects with T2DM [9]. In addition, Dapagliflozin, and Empagliflozin, two molecules belonging to the SGLT2i class, have been shown to improve clinical prognosis in patients with HFpEF regardless of T2DM [21,22,23,24]. Of interest, recently, preclinical studies also demonstrated that SGLT2i is able to reduce vascular damage and CoI in a mixed murine model of T2DM and Alzheimer’s disease [25]. Moreover, a study conducted by Mone et al. showed significant beneficial effects of Empagliflozin on cognitive and physical impairment in frail older adults with diabetes and HFpEF [15]. 

### 1.5. Aim of This Study

Thus, the aim of this study was to evaluate the effects of a six-month treatment with SGLT2i on functional, mood-related, and cognitive aspects, assessed by performing a comprehensive geriatric assessment (CGA), and on oxidative stress and platelet activation biomarkers, as possible pathophysiological targets, in a cohort of HFpEF elderly patients with T2DM.

## 2. Results

### 2.1. Baseline Characteristic of the Study Population

The study population included 115 men (76.6%) and 35 women (23.4%), with an average age of 75.8 ± 7.4 years. All patients were affected by T2DM and HFpEF. Among these, 59 patients were affected by HFimpEF; considering the associated comorbidities, 57.3% of the patients presented Ischemic Heart Disease, 25.3% presented with AF, and 22.7% had chronic obstructive pulmonary disease (COPD) (Table 1).

At baseline, 39.3% of patients were treated with sacubitril/valsartan (sac-val) therapy. In addition, at baseline, 88.0% of patients were treated with Oral antidiabetic drugs (OADs), and 69 patients were on insulin therapy. At baseline, patients had high circulating levels of markers of oxidative stress and platelet activation (Table 2).

### 2.2. Follow-Up Evaluation

**Figure 1:**
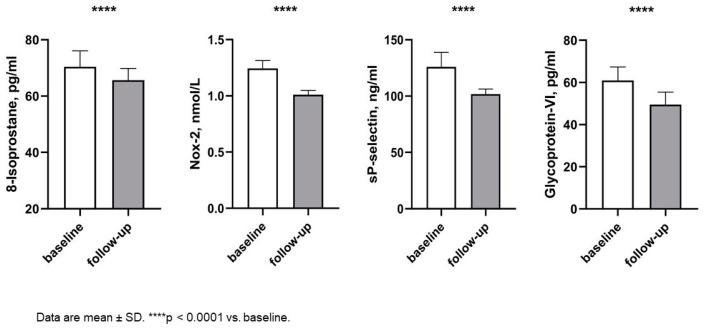
Changes in oxidative stress biomarkers and platelet activation biomarkers between baseline and follow-up. Abbreviations: Nox-2: NAPDH Oxidase 2.

After a six-month follow-up, we observed a significant improvement in respiratory rate (RR) (16.49 ± 1.66 vs. 15.78 ± 1.65 acts/min; *p* < 0.0001) and heart rate (HR) (68.63 ± 11.31 vs. 66.47 ± 10.89 beats/min; *p* = 0.002), as a consequence of hemodynamic improvement. No variations were observed in systolic and diastolic blood pressure. In addition, no change was observed in eGFR values (63.46 ± 10.41 vs. 62.30 ± 4.43 cmL/min/1.73 m^2^; *p* = 0.208) and uric acid (6.65 ± 0.24 vs. 5.62 ± 0.18 mg/dl, *p* < 0.0001), but there was no significant variation in natriemia and kaliemia. Moreover, after six months of treatment with SGLT2i, there was a significant improvement in HbA1c (7.54 ± 0.45 vs. 6.72 ± 0.41, *p* < 0.0001) and HOMA index (7.29 ± 0.89 vs. 5.47 ± 0.55; *p* < 0.0001). Of interest, at follow-up, we observed a significant improvement in oxidative stress biomarkers such as Nox-2 (1.24 ± 0.71 vs. 1.01 ± 0.04 nmol/L; *p* < 0.0001), 8-Isoprostane (70.41 ± 5.67 vs. 65.67 ± 4.16 pg/mL, *p* < 0.0001), and platelet activation biomarkers such as Sp-Selectin (125.92 ± 12.84 vs. 101.84 ± 4.42 ng/mL; *p* < 0.0001) and Gp-VI (60.99 ± 6.36 vs. 49.51 ± 5.89; *p* < 0.0001) (Figure 1).

**Figure 2:**
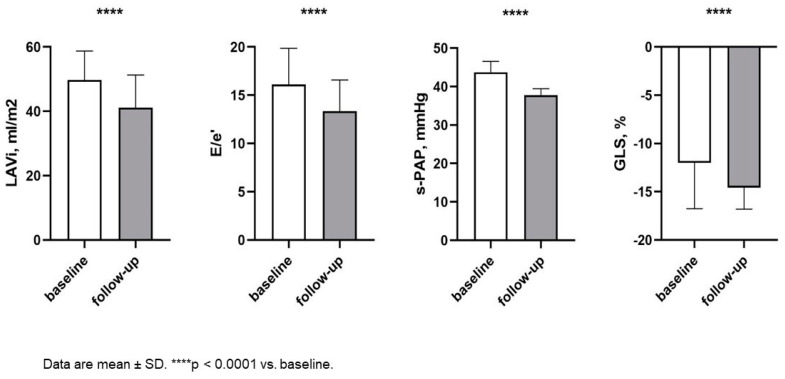
Changes in echocardiographic parameters between baseline and follow-up. Abbreviations: LAVI: left atrial volume index, E/e’: between wave E and wave e’ (reliable estimate of changes in end-diastolic blood pressure); s-PAP: systolic pulmonary arterial pressure; GLS: global longitudinal strain.

In addition, there was an important reduction in systemic and pulmonary congestion, as demonstrated by the decrease in circulating levels of NT-pro-BNP (1625 ± 292.93 vs. 738.71 ± 58.94 pg/mL; *p* < 0.0001) and in IVC diameter (19.91 ± 2.69 vs. 18.05 ± 1.66, *p* < 0.0001). Of interest, a significant improvement in left and right morpho-functional cardiac parameters was also observed (Table 3, Figure 2).

**Figure 3:**
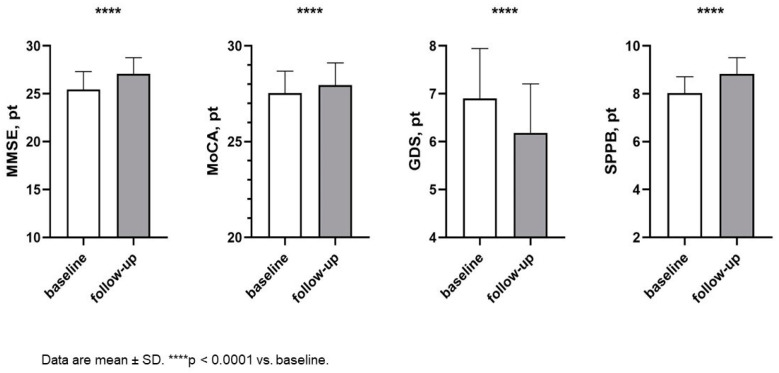
Changes in cognitive and functional parameters between baseline and follow-up. Abbreviations: MMSE: Mini-Mental state examination; MoCA: Montreal cognitive assessment; GDS: Geriatric Depression Scale; SPPB: short performance physical battery.

Finally, there was a significant improvement in MMSE (25.45 ± 1.86 vs. 27.07 ± 1.71; *p* < 0.0001) and MoCA score (27.53 ± 1.14 vs. 27.95 ± 1.16; *p* < 0.0001), with a statistically significant reduction in GDS score (6.91 ± 1.04 vs. 6.19 ± 1.02; *p* < 0.0001) and increase in SPPB (8.00 ± 0.60 vs. 8.80 ± 0.70; *p* < 0.0001) (Figure 3), indicating an amelioration in cognitive function, depressive symptoms, and functional abilities.

Of interest, a cognitive improvement was also observed in patients with CoI. In fact, based on the MMSE score, patients with CoI were 52 (34.7%) at baseline and 13 (8.7%) at follow-up (p0.016), while, according to the MoCA score, they were 35 (23.3%) at baseline and 19 (12.7%) at follow-up (*p* < 0.0001).

### 2.3. Correlation Analysis

Simple linear regression models showed that ΔMMSE correlated significantly with ΔE/e’, ΔNox-2, Δ8-Isoprostane, ΔHOMA, and ΔUricemia, while ΔMoCA correlated significantly with ΔHOMA, ΔE/e’, Δ8-Isoprostane, ΔNox-2, and ΔUricemia. Similarly, ΔGDS correlated significantly with ΔHOMA, ΔNox-2, Δ8-Isoprostane, and ΔUricemia, while ΔSPPB correlated significantly with ΔHOMA, Δ8-Isoprostane ΔNox-2, ΔE/e’, ΔUricemia (Table 4).

Considering MMSE variation as the dependent variable, ΔE/e’ was the major variable that was associated with the dependent variable for 27.2%, in the model entered also ΔNox-2, Δ8-Isoprostane, ΔHOMA, ΔUricemia, and the whole model were associated for 59.6% with MMSE variation (Table 5). When ΔMoCA was considered as the dependent variable, ΔHOMA, ΔE/e’, Δ8-Isoprostane, ΔNox-2, and ΔUricemia were associated with the dependent variable for 59.2% (Table 5).

Considering ΔGDS as a dependent variable ΔHOMA, ΔNox-2, Δ8-Isoprostane, and ΔUricemia were associated with ΔGDS for 41.6% (Table 6). Finally, for variation of SPPB, ΔHOMA was the major variable that was associated with ΔSPPB, and Δ8-Isoprostane, ΔNox-2, ΔE/e’ and ΔUricemia correlated with the dependent variable for 45.4% (Table 6).

## 3. Discussion

### 3.1. SGLT2-Inhibitors: Beyond Cardiovascular and Metabolic Benefits

The present study demonstrates that the addition of SGLT2i to the therapy of diabetic HFpEF patients significantly improves clinical and echocardiographic parameters, CGA scales, and markers of oxidative stress and platelet activation. Moreover, in view of the significant glycometabolic improvement evaluated as a reduction of HbA1c and HOMA index, oral antidiabetic drugs and insulin therapy were discontinued. The novelty of the present study is that we demonstrated the central role of SGLT2i in improving cognitive function, depressive symptoms, and functional limitations assessed by CGA, in patients with T2DM and HFpEF, as well as metabolic and echocardiographic improvement. It is well known that T2DM causes neurological complications in addition to HF, which may lead to CoI. Indeed, T2DM often causes blood-brain barrier dysfunction with altered cerebral glucose metabolism and reduced circulating brain-derived neurotrophic factor (BDNF) levels associated with CoI [26]. In view of this, it is plausible that SGLT2i therapy in elderly patients with HFimpEF and T2DM could have a beneficial effect on cognitive performance by lowering hHF and improving cardiac function.

### 3.2. Effect of SGLT2-Inhibitors on Comprehensive Geriatric Assessment and Biomarkers

In particular, recent work by Mone P. et al. demonstrated that the addition of empagliflozin to the therapy in a cohort of patients with HFpEF and T2DM resulted in a significant and greater increase in MoCA score compared with insulin and metformin after 1 month of therapy [15]. Furthermore, our previous work indicated that combining SGLT2i therapy with standard treatment for HFrEF in a group of elderly and diabetic patients led to improvements not only in physical performance but also in cognitive status and depressive symptoms. In this work, however, only 91 patients were enrolled, with a lower mean age than in this study (72.6 vs. 75.8 years), and the HFimpEF population was not included [27]. In addition, we have already demonstrated early improvement in clinical symptoms and echocardiographic parameters in elderly patients with sleep apnea syndrome and HF across the LVEF spectrum, but follow-up was limited to only 3 months [28]. In our study, the significant improvement of MMSE, MoCA, and GDS is a consequence of the improvement in cognitive function and mood disorders. In addition, functional abilities also improved, as evidenced by changes in the SPPB score. Several pathophysiological mechanisms, such as deterioration of cardiac function with frequent exacerbations and vascular abnormalities, contribute to CoI in HF, and there are also several risk factors associated with cognitive decline in patients with HF: AF, chronic subclinical inflammation, and reduced eGFR levels [15,29]. Therefore, the drugs recommended by current guidelines should be used not only to reduce hospitalizations and improve QoL but also to preserve functional abilities and cognitive function, especially in the elderly. In our study, 38.6% of patients were treated with sac-val, and the possible synergistic effects of these drugs could justify further improvement in cognitive function, in addition to an amelioration in peripheral and pulmonary congestion, followed by a reduction in the use of loop diuretics and antialdosteronic drugs [18,30,31,32]. Sac-val is safe for cognitive function, as evidenced by real-world experience and the PERSPECTIVE study [33]. Among non-CV comorbidities, depressive and anxiety syndromes may interfere with HF management. These conditions can affect up to 40% of patients but are often underdiagnosed and consequently undertreated. In addition, a recent case-control study involving several thousand individuals treated for depression, as well as subjects with the disease but without treatment, showed that diabetic patients treated with SGLT2i had a lower risk of depression than patients treated with other antidiabetic drugs [34]. On the other hand, the ability of SGLT2i to induce mild ketosis may be an additional neuroprotective mechanism that also leads to an antidepressant effect [35]. In our study, the additional administration of SGLT2i also showed a beneficial effect on depressive symptoms. In the multivariate stepwise regression model, GDS variation was strongly correlated with improvement in HOMA, CI, and Sp-selectin levels, suggesting that the positive changes in cardiac function, insulin sensitivity, and platelet biomarker activation may be responsible for the improvement of depressive symptoms. In this context, the benefit of using SGLT2i is not limited to the hemodynamic and metabolic effects already mentioned but also to the improvement of QoL and consequently depressive symptoms. A critical area of CGA that must always be assessed in patients with HF is functional ability, which can be evaluated with validated instruments such as SPPB [36]. Accordingly, the presence of physical limitations in HF represents a negative prognostic factor, as it increases the risk of hospitalization and mortality compared with patients with good functional physical ability [37]. In our study, patients’ functional abilities also improved significantly after the introduction of SGLT2i, as shown by the positive changes in SPPB. In particular, the changes in insulin sensitivity, platelet activation biomarkers, and cardiac function were associated with an improvement in SPPB.

### 3.3. Limitations and Strengths

The present study has also several limitations; firstly, it is not a randomized clinical trial, and a matched control group is not available. However, we can consider each patient under her/his own control since, before enrollment, every patient was treated with the OMT according to current guidelines but was still symptomatic. Another important limitation is represented by the relatively small sample size. Among the strengths of the present study is the enrolment of older diabetic patients with HFpEF and several comorbidities, and thus higher clinical complexity, who are often underrepresented in large randomized controlled clinical trials. In addition, we had complete and accurate clinical, hemodynamic, CGA phenotyping, and biomarker determinations of the entire study population.

## 4. Materials and Methods

### 4.1. Patients

The “MAgna GraecIa evaluation of Comorbidities in patients with Heart Failure (MAGIC-HF) study” is an observational registry that includes adult patients with HF referred to the Heart Failure Center of the Geriatric Division at the “Magna Graecia” University of Catanzaro. The registry was launched in October 2022 with the aim of evaluating not only the impact of treatment for HF and HF-related complications but also identifying new risk biomarkers involved in the pathogenesis of HF and its main associated comorbidities with a potential impact on disease treatment and prognosis. The study included outpatients suffering from chronic HF from the CATAnzaro Metabolic Risk Factors (CATAMERI) Study, an ongoing longitudinal observational study assessing cardio-metabolic risk in individuals, recruited at the University Hospital of Catanzaro. From an initial cohort of 378 patients, 119 were excluded because they were not diabetic, 45 patients were younger than 65 years, 33 had severe CKD, 18 patients showed clinically manifest dementia or severe psychiatric disorders, 13 presented Child–Pugh C liver cirrhosis and one patient was on a waiting list for cardiac transplantation. Thus, 150 consecutively recruited elderly outpatients with HFpEF and T2DM were considered for the present study. All patients presented HFpEF diagnosis according to ESC guidelines, NYHA class II-III, with stable clinical conditions and optimal medical treatment (OMT) [1]. T2DM diagnosis was defined according to American Diabetes Association (ADA) criteria or use of antidiabetic medications [38]. No patient had a clinical history of severe renal impairment with an estimated glomerular filtration rate (eGFR) < 30 mL/min/1.73 m^2^, hepatic impairment (Child–Pugh Class C), or a prior diagnosis of dementia, or severe psychiatric disorders. There were no patients selected for cardiac transplantation or electric device implantation procedures.

### 4.2. Study Design

All patients underwent a comprehensive medical history with CGA and physical examination, with anthropometric and hemodynamic parameter measurements at baseline, before the addition of SGLT2i to the therapeutic regimen, and after six months of treatment. All patients underwent a 12-lead electrocardiogram (ECG), blood chemistry tests, and a full echocardiogram-color Doppler. Relevant comorbidities and the number and type of drug therapies were also recorded in order to assess any benefit and the potential occurrence of adverse events. Evaluation of the NYHA functional class was carried out as suggested by current guidelines, and QoL assessment was performed using the Minnesota Living with Heart Failure Questionnaire (MLHFQ) [39].

#### 4.2.1. Echocardiograms

Echocardiographic recordings were made using a VIVID E-95 ultrasound system (GE Technologies, Milwaukee, WI, USA) equipped with a 2.5 MHz transducer. All patients were examined at rest and in left lateral decubitus. Measurements were obtained according to the recommendations of the American Society of Echocardiography [40].

#### 4.2.2. Laboratory Measurements and Oxidative Stress and Platelets Activation Biomarkers Serum Levels

All laboratory measurements were performed after at least 12 h of fasting on peripheral blood samples. Blood samples were collected in tubes with separator gel and centrifuged at 4000 rpm for 15 min to obtain serum samples that were immediately stored at −80 °C. ELISA kits were employed to detect serum levels of 8-isoprostane (Cayman Chemical, Ann Arbor, MI, USA); NAPDH-oxidase 2 (Nox-2); and Sp-selectin (both from MyBioSource, San Diego, CA, USA). Values of 8-isoprostane were expressed as pg/mL; the lower detection limit of the assay was 0.8 pg/mL; the inter-assay coefficient of variation (CoV) was <9.6%; and the intra-assay CoV was <19.9%. Values of Nox-2 were expressed as nmol/L, the lower detection limit of the assay was 0.25 nmol/L; the intra-assay CoV was <9%; and the inter-assay CoV was <11%. Finally, Sp-selectin concentrations were expressed in ng/mL; the kit had a lower detection limit of 15 ng/mL; the intra-assay CoV was <10%; and the inter-assay CoV was <15% [41].

#### 4.2.3. Comprehensive Geriatric Assessment

At baseline before the introduction of SGLT2i and after a six-month follow-up, all patients underwent a CGA. Specifically, cognitive function was assessed with the Mini-Mental State Examination (MMSE) and the Montreal Cognitive Assessment (MoCA) [42,43], in addition, the presence of depressive symptoms was estimated with the Geriatric Depression Scale (GDS) [44], finally, frailty was evaluated with the Short physical performance battery (SPPB) [45].

#### 4.2.4. Ethics Committee

The study was approved by the local Institutional Ethics Committees of the University “Magna Graecia” of Catanzaro (code protocol number 2012.63) for CATAnzaro Metabolic Risk factors (CATAMERI) Study; and by Comitato Etico “Area Centro” (2022.384) for the “MAgna GraecIa evaluation of Comorbidities in patients with Heart Failure (MAGIC-HF) study”; ClinicalTrials.gov Identifier: NCT05915364. All investigations were made in accordance with the principles of the Declaration of Helsinki. The studies were conducted in accordance with the local legislation and institutional requirements. The participants provided their written informed consent to participate in this study.

#### 4.2.5. Statistical Analysis

Continuous variables are expressed as mean and standard deviation (SD) (normally distributed data) or as the median and interquartile range (IQR) (non-normally distributed data). Categorical data are expressed as numbers and percentages. The evolution of therapies over time was assessed with the χ2 test. Longitudinal changes in key variables at follow-up were analyzed with the *t*-test or Wilcoxon’s test for paired data, and comparisons between the two groups were made with the *t*-test and Mann–Whitney test for unpaired data when appropriate. A simple linear regression analysis was performed to assess the correlation between the change in MMSE, MoCA, GDS, and SPPB values, expressed as (Δ) between baseline and follow-up (ΔT0-6), and the change in several covariates, also expressed as ΔT0-6. Variables that reached statistical significance were entered into a stepwise multivariate linear regression model to assess the magnitude of their individual effects on ΔMMSE, MoCA, GDS, and SPPB. Differences were considered significant at *p* < 0.05. Statistical analysis was carried out using the SPSS V20.0 program for Windows (SPSS Inc., Chicago, IL, USA).

## 5. Conclusions

This real-life study carried out in a cohort of elderly patients suffering from chronic HFpEF and T2DM with several comorbidities, demonstrated that treatment with SGLT2i for six months induced important improvements in clinical, metabolic, and hemodynamic outcomes. These changes were associated with an improvement in cognitive, mood, and functional status, as well as markers of oxidative stress and platelet activation in the whole population. In conclusion, in elderly patients suffering from HFpEF and T2DM, treatment with SGLT2i results in important clinical benefits affecting not only cardiovascular and metabolic aspects but also CGA scales with significant improvement in QoL.

## Figures and Tables

**Table 1:** Clinical characteristics and pharmacological therapies of the study population at baseline.

All Population (n. 150)	
Age, years	75.8 ± 7.4
Male gender, n ( %)	115 (76.6)
HFimpEF, n (%)	59 (39.3)
IHD, n (%)	86 (57.3)
VHD, n (%)	80 (53.3)
AF, n (%)	38 (25.3)
T2DM, n (%)	150 (100)
AH, n (%)	96 (64.0)
COPD, n (%)	34 (22.7)
Dyslipidaemia, n (%)	58 (38.7)
CKD, n (%)	42 (28.0)
Smokers, n (%)	58 (38.7)
ARNI, n (%)	59 (39.3)
ACEi/ARB, n (%)	91 (60.7)
Loop Diuretics, n (%)	125 (83.3)
MRAs, n (%)	79 (52.7)
β-blockers, n (%)	77 (51.3)
OACs, n (%)	34 (22.7)
Anti-platelet drugs, n (%)	78 (52.0)
Statins, n (%)	78 (52.0)
OADs, n (%)	132 (88.0)
Insulin therapy, n (%)	69 (46.0)

Abbreviations: HFimpEF: Heart failure improved ejection fraction; IHD: Ischemic Heart Disease; VHD: Valvular Heart Disease, AF: atrial fibrillation; T2DM: Diabetes Mellitus Type 2; AH: Arterial Hypertension, COPD: chronic obstructive pulmonary disease; CKD: chronic kidney disease; ARNI: angiotensin receptor neprilysin inhibitor; ACEi: Angiotensin-converting enzyme inhibitors; ARB: Angiotensin II receptor blockers; MRAs: Mineralocorticoid receptor antagonists; OACs: Oral anti-coagulants; OADs: oral anti-diabetic drugs.

**Table 2:** Clinical and demographic characteristics of the study population at baseline and follow-up.

All Population (n. 150)	Baseline	Follow-Up	*p* *
MMSE, pt	25.45 ± 1.86	27.07 ± 1.71	<0.0001
MoCA, pt	27.53 ± 1.14	27.95 ± 1.16	<0.0001
GDS, pt	6.91 ± 1.04	6.19 ± 1.02	<0.0001
SPPB, pt	8.00 ± 0.60	8.80 ± 0.70	<0.0001
BMI, Kg/m^2^	32.20 ± 4.90	31.38 ± 4.86	<0.0001
SBP, mmHg	125.18 ± 15.13	125.15 ± 15.68	0.967
DBP, mmHg	73.95 ± 9.94	75.02 ± 8.43	0.240
HR, beats/min	68.63 ± 11.31	66.47 ± 10.89	0.002
RR, acts/min	16.49 ± 1.66	15.78 ± 1.65	<0.0001
Hb, g/dL	12.13 ± 1.59	12.84 ± 1.42	<0.0001
HCT, (%)	36.47 ± 5.90	39.12 ± 5.41	<0.0001
PLT, μ/μL × 10^3^	200.95 ± 47.92	212 ± 36.73	0.001
Na, mEq/L	139.69 ± 5.03	140.08 ± 3.33	0.423
K, mEq/L	4.45 ± 0.40	4.44 ± 0.34	0.859
HOMA, pt	7.29 ± 0.89	5.47 ± 0.55	<0.0001
LDL, mg/dL	75.09 ± 36.57	61.72 ± 29.58	<0.0001
HDL, mg/dL	45.68 ± 11.76	47.33 ± 17.53	0.300
Triglycerides, mg/dL	134.31 ± 50.10	125.81 ± 38.58	0.008
Uricemia, mg/dL	6.65 ± 0.24	5.62 ± 0.18	<0.0001
hs-CRP, mg/L	5.33 ± 0.29	4.15 ± 0.27	<0.0001
Creatinine, mg/dL	1.14 ± 0.137	1.13 ± 0.44	0.947
eGFR, mL/min/1.73 m^2^	63.46 ± 10.41	62.30 ± 4.43	0.208
NT pro BNP, pg/dL	1625 ± 292.93	738.71 ± 58.94	<0.0001
HbA1c, %	7.54 ± 0.45	6.72 ± 0.41	<0.0001
8-Isoprostane, pg/mL	70.41 ± 5.67	65.67 ± 4.16	<0.0001
Nox-2, nmol/L	1.24 ± 0.71	1.01 ± 0.04	<0.0001
Sp-selectin, ng/mL	125.92 ± 12.84	101.84 ± 4.42	<0.0001
Gp-VI, pg/mL	60.99 ± 6.36	49.51 ± 5.89	<0.0001

* Performed by *t*-test for unpaired data. Abbreviations: MMSE: Mini-mental state examination; MoCA: Montreal Cognitive Assessment; GDS: Geriatric Depression Scale; SPPB: Short Performance physical battery; BMI: Body mass index; SBP: Systolic blood pressure; DBP: Diastolic blood pressure; HR: Heart rate; RR: Respiratory rate; Hb: Hemoglobin; HTC: Hematocrit; PLT: Platelets; Na: sodium; K: potassium; HOMA: Homeostasis Model Assessment; LDL: Low-density lipoprotein; HDL: high-density lipoprotein; hs-CRP: high sensitivity C-Reactive protein; e-GFR: estimate glomerular filtration rate; HbA1c: glycated hemoglobin; Nox-2: NADPH oxidase 2; Gp-VI: Glycoprotein VI.

**Table 3:** Echocardiographic characteristics of the study population at baseline and follow-up.

All Population (n. 150)	Baseline	Follow-Up	*p* *
LAVi, mL/min	49.71 ± 9.00	41.11 ± 10.16	<0.0001
LVEDV/BSA, mL/m^2^	87.39 ± 5.60	84.78 ± 4.64	<0.0001
LVESV/BSA, mL/m^2^	49.77 ± 3.69	46.41 ± 3.23	<0.0001
LVEF, %	43.05 ± 2.05	45.25 ± 2.44	<0.0001
Cardiac Index, mL/min/m^2^	2061.75 ± 78.26	2122.17 ± 100.59	<0.0001
GLS, %	−12.01 ± 4.74	−14.57 ± 2.23	<0.0001
E/A	0.93 ± 0.51	0.93 ± 0.42	0.842
E/e’	16.11 ± 3.75	13.37 ± 3.24	<0.0001
RAA, cm^2^	19.46 ± 6.92	17.29 ± 4.16	<0.0001
RVOTp, cm	2.59 ± 0.36	2.41 ± 0.30	<0.0001
TAPSE, mm	17.87 ± 1.11	20.33 ± 1.62	<0.0001
s-PAP, mmHg	43.71 ± 2.85	37.76 ± 1.69	<0.0001
TAPSE/s-PAP, mm/mmHg	0.41 ± 0.38	0.54 ± 0.48	<0.0001
IVC, mm	19.91 ± 2.69	18.05 ± 1.66	<0.0001

* Performed by *t*-test for unpaired data. Abbreviations: LAVI: left atrial volume index; LVEDV/BSA: left ventricular end-diastolic volume index/body surface area; LVESV/BSA: left ventricular end-systolic volume index/body surface area; LVEF: left ventricular ejection fraction; GLS: global longitudinal strain; E/A: the ratio between wave E (the wave of rapid filling in early diastole) and wave A (the wave of atrial contraction); E/e’: between wave E and wave e’ (reliable estimate of changes in end-diastolic blood pressure); RAA: Right Atrium Area; RVOTp: Right Ventricular Outflow Tract proximal; TAPSE: Tricuspid annular plane systolic excursion; s-PAP: systolic pulmonary arterial pressure; IVC: inferior vena cava.

**Table 4:** Simple linear correlation analysis between Δ of MMSE, MoCA, GDS, and SPPB and Δ of different covariates in the study population.

	ΔMMSE	ΔMoCA	ΔGDS	ΔSPPB
	R/P	R/P	R/P	R/P
ΔE/e’	−0.316/<0.0001	−0.334/<0.0001	0.097/0.191	−0.255/<0.0001
ΔGLS, %	0.044/0.440	0.025/0.664	−0057/0.404	−0.036/0.588
ΔNox-2, nmol/L	−0.284/<0.0001	−0.186/0.001	0.211/0.003	0.228/0.001
ΔGp-VI, pg/mL	−0.014/0.820	−0.013/0.814	0.034/0.605	0.042/0.501
Δ8-Isoprostane, pg/mL	−0.265/<0.0001	−0.252/<0.0001	0.152/0.036	0.033/<0.0001
ΔSp-selectin, ng/mL	−0.003/0.957	0.013/0.822	−0.041/0.547	−0.036/0.582
ΔHbA1c, %	0.0.19/0.722	0.021/0.707	−0.016/0.811	0.009/0.885
ΔHOMA, pt	−0.230/<0.0001	−0.326/<0.0001	0.395/<0.0001	−0.263/<0.0001
ΔNT pro BNP, pg/dL	−0.055/0.323	−0.057/0.303	0.041/0.538	−0.065/0.309
Δhs-CRP, mg/L	−0.035/0.522	−0.045/0.418	−0.033/0.619	−0.018/0.776
ΔUricemia, mg/dL	0.169/0.003	0.145/0.011	−0.166/0.015	0.128/0.051

Abbreviations: Δ: variation between baseline and six-month follow-up, MMSE: Mini Mental state examination; MoCA: Montreal cognitive assessment; GDS: Geriatric Depression Scale; SPPB: short performance physical battery; E/e’: between wave E and wave e’ (reliable estimate of changes in end-diastolic blood pressure); GLS: global longitudinal strain; Nox-2: NAPDH Oxidase 2; Gp-VI: Glycoprotein VI, HbA1c: glycated hemoglobin; HOMA: homeostatic model assessment; hs-CRP: high sensitivity C-reactive protein.

**Table 5:** Stepwise multivariate linear analysis between Δ of MMSE and Δ of MoCA as dependent variable and Δ of different covariates.

Δ of MMSE as Dependent Variable				Δ of MoCA as Dependent Variable			
All	R^2^ Partial	R^2^ Total	*p*	All	R^2^ Partial	R^2^ Total	*p*
ΔE/e’	27.2	27.2	<0.0001	ΔHOMA, pt	29.5	29.5	<0.0001
ΔNox-2, nmol/L	17.3	44.5	<0.0001	ΔE/e’	17.9	47.4	<0.0001
Δ8-Isoprostane, pg/mL	6.8	51.3	<0.0001	Δ8-Isoprostane, pg/mL	5.4	52.8	<0.0001
ΔHOMA, pt	5.5	56.8	<0.0001	ΔNox-2, nmol/L	4.5	57.3	<0.0001
ΔUricemia. %	2.8	59.6	0.001	ΔUricemia. %	1.9	59.2	0.006

Abbreviations: Δ: variation between baseline and six-month follow-up. MMSE: Mini-Mental state examination; MoCA: Montreal cognitive assessment; E/e’: between wave E and wave e’ (reliable estimate of changes in end-diastolic blood pressure); Nox-2: NAPDH Oxidase 2; HOMA: homeostatic model assessment.

**Table 6:** Stepwise multivariate linear regression analysis between Δ of GDS and Δ of SPPB as the dependent variable and Δ of different covariates.

Δ of GDS as Dependent Variable				Δ of SPPB as Dependent Variable			
All	R^2^ Partial	R^2^ Total	*p*	All	R^2^ Partial	R^2^ Total	*p*
ΔHOMA, pt	29.8	29.8	<0.0001	ΔHOMA, %	21.3	21.3	<0.0001
ΔNox-2, nmol/L	5.5	35.3	<0.0001	Δ8-Isoprostane, pg/mL	12.4	33.7	<0.0001
Δ8-Isoprostane, pg/mL	3.4	38.7	0.003	ΔNox-2, nmol/L	5.3	39.0	<0.0001
ΔUricemia, %	2.9	41.6	0.005	ΔE/e’	5.1	44.1	<0.0001
---	---	---	---	ΔUricemia, %	1.3	45.4	0.033

Abbreviations: Δ: variation between baseline and six-month follow-up. GDS: Geriatric Depression Scale. SPPB: short-performance physical battery. E/e’: between wave E and wave e’ (reliable estimate of changes in end-diastolic blood pressure); Nox-2: NAPDH Oxidase 2; HOMA: homeostatic model assessment.

## Data Availability

The raw data supporting the conclusions of this article will be made available by the authors, without undue reservation.
